# Vasovagal syncope related to pain procedures in a pain clinic at a tertiary Lebanese hospital between 2016 and 2019

**DOI:** 10.1186/s13104-021-05541-8

**Published:** 2021-04-13

**Authors:** Sara Kamar, Souheil Hallit, Souheil Chamandi

**Affiliations:** 1 Faculty of Medicine and Medical Sciences, Holy Spirit University of Kaslik (USEK), Jounieh, Lebanon; 2 INSPECT-LB: National Institute of Public Health, Clinical Epidemiology and Toxicology, Beirut, Lebanon; 3 Department of Anesthesiology, Notre Dame des Secours University Hospital, Byblos, Lebanon

**Keywords:** Pain procedure, Vasovagal syncope, Fainting, Blood pressure, Heart rate

## Abstract

**Objectives:**

Our study focuses on evaluating the factors associated with vasovagal syncope (VVS) when having a pain procedure at the pain clinic as well as showing variation in vital signs associated with fainting across different periods of the procedure (before, during and after).A retrospective case control study was conducted in a university hospital in Lebanon (CHU-NDS) on adult Lebanese patients with data taken from the archives covering a 4 year period (2016–2019).

**Results:**

The multivariable analysis showed that a higher systolic blood pressure per-procedure was significantly associated with lower odds of having vasovagal syncope. An adequate dose of a vasopressor like ephedrine can be used to prevent a vasovagal event from happening. In our study the blood pressure component was more significant than the heart rate component which stayed in the normal range limit in the three different periods of procedure. Cases having a pain procedure for the first time represent 59.6% of the occurrence of VVS. Vasovagal syncope is a complication that can be prevented in high risk patients. Our study suggests taking preventive measures for VVS for patients with first time infiltration status especially if appearing in an anxious state.

## Introduction

Of the many tools available to identify and treat the pain generator, interventional therapy often is at the top of the list for most pain specialists and it can be particularly useful for back pain sufferers. Indeed, multiple therapeutic spinal interventional techniques are applied in managing chronic back pain. Nevertheless, safe practice of interventional pain management requires knowledge of not only how to perform a procedure but also an understanding of the potential per procedural complications. In fact, a vasovagal reaction is a complex neurovascular reflex mediated by the efferent component of the autonomic nervous system, and it is one of the most common cardiovascular complications that can occur during an interventional procedure [1].

The term “vaso-vagal” was first used by Sir William Gowers in 1907 to describe a constellation of “vagal” symptoms, including epigastric, respiratory, and cardiac discomfort occurring in association with vasomotor spasm. Sir Thomas Lewis, a British cardiologist, redefined vasovagal syncope along pathophysiological lines of a fall in blood pressure as an added phenomenon to a slowing in ventricular rate in 1932 [2].

Many studies were done regarding VVS related to invasive procedures like for example blood donation [3–5] but very few approached fainting in the setting of pain procedures. A previous study done regarding the effects of lumbar spinal nerve analgesia on the cardiovascular system [6] showed a minor rise in systolic and diastolic blood pressure, heart rate as well as a normalization of these parameters after the nerve root infiltration. These findings were similar for patients with and without pre-existing cardiovascular diseases. 5 of 117 patients having sciatic pain suffered from presyncope after the nerve root infiltration and these individuals were found to be significantly younger than those without presyncope. Finally presyncope was more frequent during their first treatment with lumbar paravertebral nerve root infiltration in comparison to repeated application of this therapy [6].

Patients who endure infiltration for their back or cervical pains are subjected to psychological and hemodynamic challenges during the process. Even though VVS is a transient loss of consciousness with complete spontaneous recovery, it often has a debilitating and uneasy effect [7]. Our study focuses on evaluating the factors associated with VVS regarding interventional procedures specifically at the pain clinic, showing variation in vital signs associated with fainting across different periods of the procedure thus suggesting variation in the underlying mechanisms leading to VVS, identifying high risk patients and consequently taking adequate measures to prevent this complication.

## Main text

### Methods

#### Study design

A retrospective case control study was conducted in a university hospital in Lebanon (CHU-NDS) on adult Lebanese patients with data taken from the archives covering a 4 year period (2016–2019). It included 188 patients: 94 cases representing all the ones who had endured a VVS or presyncope related to a pain procedure during this time frame and 94 controls with matching demographic features and without occurrence of VVS or related symptoms. The patients from the control group, having matching age and sex, were identified, analogous data inserted on an excel sheet and were randomly selected using an online software.

#### Ethical approval

The ethics committee of the hospital approved the study protocol.

#### Data collection

Data were collected from the medical file of each patient, gathering donor demographic information (age, sex), biometric characteristics (weight), medical background (hypertension, diabetes, dyslipidemia) with applicable treatments (ACE inhibitors, ARBs, CCB, diuretics, betablockers) and clinical measurements (heart rate and blood pressure prior, per and after infiltration). Additionally, the psychological state of the patients was identified before the interventional procedure (calm, more or less calm, anxious).Furthermore, the infiltration status and its relation to occurrence of syncope were noted as well as the use of ephedrine prior to the procedure.

#### Statistical analysis

The SPSS software v.25 was used for all statistical analyses. The Chi-square or the Fisher exact tests were used to compare categorical variables between cases and controls, whereas the Student t-test was used to compare between continuous variables. A forward logistic regression was conducted taking the presence/absence of vasovagal syncope as the dependent variable and taking all variables as independent ones. The Nagelkerke R^2^ value was calculated provide an indication of the amount of variation in the dependent variable explained by the model; the higher the R^2^ value, the more the independent variables explain the dependent one. p < 0.05 was considered statistically significant.

### Results

#### Sociodemographic characteristics

For the four year study period, there were 94 cases that endured VVS and 94 controls that were picked randomly with matching demographic status. The results showed that among the participants 52.1% were males, with a median age of 45.16 ± 11.42, and a median weight of 78.37 ± 15.37.

#### Comparison between cases and controls

A significantly lower mean systolic and diastolic blood pressure pre-procedure, per-procedure, post-procedure, and heart rate per-procedure were found in patients who had a vasovagal syncope compared to those who did not (Table 1).

**Table 1:** Bivariate analysis of factors associated with the presence/absence of vasovagal syncope

Variable	Absence of vasovagal syncope (N = 94)	Presence of vasovagal syncope (N = 94)	*p*
Sex			0.884
Male	48 (51.1%)	48 (51.1%)	
Female	46 (48.9%)	46 (48.9%)	
Medical history (any disease)			0.374
No	58 (61.7%)	52 (55.3%)	
Yes	36 (38.3%)	42 (44.7%)	
Hypertension			0.854
No	75 (79.8%)	76 (80.9%)	
Yes	19 (20.2%)	18 (19.1%)	
Dyslipidemia			0.468
No	77 (81.9%)	73 (77.7%)	
Yes	17 (18.1%)	21 (22.3%)	
Diabetes			1
No	86 (91.5%)	86 (91.5%)	
Yes	8 (8.5%)	8 (8.5%)	
Other diseases			0.756
No	89 (94.7%)	88 (93.6%)	
Yes	5 (5.3%)	6 (6.4%)	
Beta blockers			**0.044**
No	84 (89.4%)	91 (96.8%)	
Yes	10 (10.6%)	3 (3.2%)	
ACE inhibitors			0.7
No	91 (96.8%)	90 (95.7%)	
Yes	3 (3.2%)	4 (4.3%)	
ARBs			0.516
No	88 (93.6%)	90 (95.7%)	
Yes	6 (6.4%)	4 (4.3%)	
Calcium channel blockers			0.246
No	94 (100%)	91 (96.8%)	
Yes	0 (0%)	3 (3.2%)	
SSRIs			0.516
No	90 (95.7%)	88 (93.6%)	
Yes	4 (4.3%)	6 (6.4%)	
Diuretics			0.246
No	94 (100%)	91 (96.8%)	
Yes	0 (0%)	3 (3.2%)	
Psychological state			0.077
Calm	57 (60.6%)	45 (47.9%)	
More or less calm	23 (24.5%)	38 (40.4%)	
Anxious	14 (14.9%)	11 (11.7%)	
Infiltration status and occurrence of syncope			**< 0.001**
First time/repeat infiltration, no syncope	94 (100%)	0 (0%)	
First time infiltration with syncope	0 (0%)	56 (59.6%)	
Repeat infiltration and Ephedrine intake, no syncope	0 (0%)	15 (16.0%)	
Repeat infiltration with syncope	0 (0%)	23 (24.5%)	
Age (in years)	45.28 ± 11.31	45.16 ± 11.42	0.944
Weight (in Kg)	79.16 ± 17.58	78.37 ± 15.37	0.744
Systolic blood pressure pre-procedure (mm Hg)	12.17 ± 1.63	11.11 ± 1.85	**< 0.001**
Diastolic blood pressure pre-procedure (mm Hg)	7.17 ± 0.90	6.79 ± 0.87	**0.004**
Systolic blood pressure per-procedure (mm Hg)	12.04 ± 1.45	9.32 ± 1.48	**< 0.001**
Diastolic blood pressure per-procedure (mm Hg)	7.14 ± 0.85	6.15 ± 0.88	**< 0.001**
Systolic blood pressure post-procedure(mm Hg)	12.09 ± 1.51	10.72 ± 1.62	**< 0.001**
Diastolic blood pressure post-procedure (mm Hg)	7.18 ± 0.86	6.68 ± 0.88	**< 0.001**
Heart rate pre-procedure (beats/min)	77.43 ± 9.63	75.98 ± 9.28	0.296
Heart rate per-procedure (beats/min)	77.02 ± 8.03	72.43 ± 10.90	**0.001**
Heart rate post-procedure (beats/min)	77.02 ± 8.13	75.79 ± 9.06	0.327

Numbers in bold indicate significant *p*-values

#### Multivariable analysis

The results of the logistic regression taking the presence/absence of vasovagal syncope as the dependent variable, showed that a higher systolic blood pressure per-procedure was significantly associated with lower odds of having vasovagal syncope (aOR = 0.28; p < 0.001; 95% 0.20–0.40) (Table 2).

**Table 2:** Multivariable analysis: logistic regression taking the presence/absence of vasovagal syncope as the dependent variable

Variable	*p*	aOR	95% CI
Systolic blood pressure per-procedure	< 0.001	0.28	0.20–0.40

Nagelkerke R^2^ = 60.7%

### Discussion

During the pain procedure, the mean systolic and diastolic blood pressure values were found to be 9.32 ± 1.48 mmHg and 6.15 ± 0.88 mmHg respectively. In fact, the multivariable analysis showed that a higher systolic blood pressure per-procedure was significantly associated with lower odds of having vasovagal syncope. This can be explained by the effects of substances used during spinal infiltration: local anesthetics primarily function by reversibly blocking the sodium channels in nerve and muscle membranes having a direct effect on sympathetic nerves when injected into the subarachnoid space which may induce hypotension and the cardiac tissue when injected intravascularly which may result in reduced contractility [8]. In addition corticosteroid preparations are also used and the most common ones being methylprednisolone, triamcinolone, and betamethasone. Side effects are uncommon but include transient hypotension. Supplemental fluids are important during most procedures whether it is a high risk procedure or not. When the patient has been NPO for 3 h or particularly during morning procedures when they have been NPO since the night before, they are somewhat volume depleted and are more prone to vasovagal reactions. Supplemental fluids before, during, and after the procedure help prevent such reactions. In addition, it is helpful to have fluids already flowing in the event that the patient becomes hypotensive or to help flush medications through the line. Supplemental fluids should be used cautiously if the patient is volume-sensitive such as in congestive heart failure or renal pathology [8].

In our study the blood pressure component was more significant than the heart rate component which stayed in the normal range limit in the three different periods of infiltration despite the fact that it was lower during procedure phase compared to the other phases.

Furthermore, an adequate dose of a vasopressor like ephedrine can be used to prevent a vasovagal event from happening. Since vasovagal episodes are preceded by various symptoms including light‐headedness, dizziness, nausea, sweating, pallor, unclear thinking and visual disturbances it can be prevented by a dose of ephedrine if the patient presents those symptoms clinically or if he has a history of VVS in previous interventional procedures. As showed in our study, 15 patients coming for repeated infiltration with a history of VVS and after receiving an adequate dose of ephedrine did not experience syncope. Indeed, whether the patient endures a vasovagal syncope or presyncope symptoms or comes for repeat pain procedure and needs a prophylactic dose of ephedrine, the starting dose used is 5 mg per milliliters given intravenously and is re-administered as needed.

This group of patients included 7 cases with no medical history, 1 with auto immune disease, 1 with dysthyroidism, 3 having dyslipidemia and 3 others with diabetes mellitus knowing that a patient was both diabetic and dyslipidemic and finally one with hypertension. Our study showed that the latter is one out of the 18 patients with a history of hypertension that did not suffer from syncope after receiving a dose of ephedrine prior to the procedure. Indeed, syncope occurred during his first neuraxial block and consequently a prophylactic dose of ephedrine was used when he showed up for a second time leading to a stable blood pressure throughout the intervention and an absence of a vasovagal reaction.

In addition, infiltration status concerning a procedure is an important factor to take into consideration. A study done by Hanefeld et al. found that presyncope was more frequent during the first treatment with lumbar paravertebral nerve root infiltration in comparison to repeated application of this therapy [6]. Our study also showed similar results with cases having had an infiltration for the first time representing 59.6% of the occurrence of VVS. In addition, a study done by Bravo et al. suggested that in the pre-donation phase the risk factors are mainly demographic characteristics of donors (young age and first‐time status) and the stimuli for fainting may be primarily psychological [9]. In our study, concerning spinal infiltration, the use of SSRIs was slightly higher in cases and only 47.9% of them were calm during admission. As for the age factor, having a median lifespan of cases around 45.16 ± 11.42, the study included a low number of young patients since back pain has a higher incidence with age.

A study done by Deacon et al. treating fear of needles and vasovagal reactions among phlebotomy patients concluded that a subgroup of approximately 2% of phlebotomy patients endorsed significant concerns about injections. Among these concerns, a probable diagnosis of needle phobia and being disgusted by needles were strongly associated with adverse reactions to injections. The use of needles is an important factor associated with VVS [10]. This can be the cause of increased anxiousness hence the cause of syncope in the pre- and per procedurephase.

### Conclusion

Enduring an interventional procedure combines both physical and psychological challenges. Pain procedures like spinal infiltrations are interventional techniques used for back and cervical pain but even though complications of these injections are rare awareness is essential. Vasovagal syncope is a complication that can be prevented in high risk patients. Because some intervention measures have been shown to be effective in reducing the risk of fainting reactions, it becomes important to evaluate the characteristics of the donors most likely to react and the intervention period with the highest risk of occurrence of VVS so that appropriate interventions can be selected for reactions in each period. In consequence, the results of our study suggest taking preventive measures for VVS for first time infiltration status especially if appearing in an anxious state. This reaction can largely be avoided with gentle handling of the patient and proper counseling and adequate preventive ephedrine can be used. In addition, ephedrine is to be used in a positive history of VVS in previous interventional procedures or in the occurrence of presyncope. Since VVS is likely to occur during an infiltration due to a lower blood pressure a slow and careful injection technique is to be used as well as fluid supplementation.

## Limitations

Our study focused on a sample of patients taken from one hospital only and is not representative of the whole Lebanese population. Moreover, a residual confounding bias is possible since we included the demographic, medical, hemodynamic and psychological aspects related to a VVS related to a pain procedure; however more factors could be also related for the study of its occurrence. To add up, we should note the possibility that our results underestimate the true point prevalence of vasovagal syncope due to the unique characteristics of our sample. VVS has two peak incidences: one between ages 10 and 30 years, with a median peak around 15 years and the other after 70 years. Nevertheless, back or cervical pain is most likely to occur in adult patients excluding teenagers. Finally, the lack of electrocardiograms in patients enduring VVS cannot exclude cardiogenic syncope.

## Data Availability

There is no public access to all data generated or analyzed during this study to preserve the privacy of the identities of the individuals. The dataset that supports the conclusions is available to the corresponding author upon request.

## Abbreviations

VVS: Vasovagal syncope
HUT: Head upright tilt test
SSRI: Selective Serotonin Reuptake Inhibitor
ACE: Angiotensin-converting-enzyme
ARB: Angiotensin receptor blocker
NPO: Nil per os (nothing by mouth)
